# Executive Dysfunction in MCI: Subtype or Early Symptom

**DOI:** 10.1155/2012/936272

**Published:** 2012-05-30

**Authors:** Ivar Reinvang, Ramune Grambaite, Thomas Espeseth

**Affiliations:** ^1^Center for the Study of Human Cognition, Department of Psychology, University of Oslo, 0316 Oslo, Norway; ^2^Center for Advanced Study, Norwegian Academy of Science and Letters, 0271 Oslo, Norway; ^3^Department of Neurology, Akershus University Hospital, 1478 Lørenskog, Norway; ^4^Faculty Division of Psyciatry, Division of Mental Health and Addiction, Oslo University Hospital, 0407 Oslo, Norway; ^5^Department of Biological and Medical Psychology, Faculty of Psychology, University of Bergen, 5020 Bergen, Norway

## Abstract

Mild cognitive impairment (MCI) may take several forms, and amnestic MCI (aMCI) has been recognized as an early stage of Alzheimer's Disease (AD). Impairment in executive functions including attention (eMCI) may be indicative of several neurodegenerative conditions. Executive impairment is frequently found in aMCI, it is significant for prognosis, and patients with eMCI may go on to develop AD. Recent studies have found changes in white matter integrity in patients with eMCI to be more sensitive than measures of cortical atrophy. Studies of genetic high-risk groups using sensitive cognitive neuroscience paradigms indicate that changes in executive function may be a cognitive marker useful for tracking development in an AD pathophysiological process.

## 1. Introduction

The diagnosis of MCI due to AD (the symptomatic predementia phase of AD) has been recently proposed by the National Institute on Aging and the Alzheimer's Association [1]. Diagnostic criteria include concern regarding a change in cognition, impairment in one or more cognitive domains, preservation of independence in functional abilities, and not demented. Within the generally accepted framework, clinical presentations may be in the memory (amnestic) or nonmemory domains [2].

Clinical studies of prodromal stages to AD tend to focus on persons with amnestic MCI (aMCI). Both the Alzheimer's Disease Neuroimaging Initiative (ADNI) and the Mayo clinic cohorts typify this population. In the Mayo clinical study of aging, persons in the age range of 70–89 years are enrolled, with 2/3 of the MCI group being aMCI [3]. ADNI [4] enrolls participants between ages 55 and 90, and mean age for the MCI group is 74.7 years. Researchers from the Mayo clinic proposed quantitative criteria for identifying MCI as a prodromal stage to Alzheimer's disease in 1999 [5]. They stressed the importance of memory impairment and proposed quantitative criteria specifying the level of memory deficit relative to global cognitive functioning. In ADNI, MCI is defined as a Clinical Dementia Rating of 0.5, and performance on the free recall measure of a neuropsychological memory test (Logical Memory II) is below a given threshold. Since the vast majority of the subjects characterized as aMCI will develop AD, aMCI may be defined as a prodromal condition of AD [6]. The pathophysiological basis and prognosis of nonamnestic MCI remains unclear, and the group is probably heterogenous [6, 7], including patients with frontotemporal dementia [8], Parkinson's disease [9], dementia with Lewy bodies [10], vascular dementia [11], and neuropsychiatric conditions (depression) [12]. Change in the frontostriatal network supporting executive functions may occur as a part of healthy aging [13, 14]. Thus, an MCI subgroup with isolated executive difficulties may be an extreme group of normal aging.

The objective of this paper is to review executive/attentional impairment as an important aspect of MCI or pre-MCI in terms of symptom manifestation and importance for disease progression. We will focus on recent research on MRI and genetic markers that may serve to further understanding of the pathophysiological processes underlying executive MCI (eMCI) and its relation to AD.

## 2. Executive Dysfunction in MCI

Attention and executive impairment are frequent and disabling symptoms in MCI when measured with neuropsychological tests ranging from simple processing speed tasks to tasks of complex problem solving. The distinction between clinical tests of attention and test of executive function is a fuzzy one and they are here treated as on the same continuum. There is no consensus on how executive function should be tested in clinical studies [15], and studies that take into consideration developments in cognitive psychology [16] find high frequency of executive impairment in both amnestic and nonamnestic MCI [17], with some subfunctions more affected than others.

Alzheimer's disease (AD) is the most common cause of dementia and accounts for approximately 60–70% of all dementia cases [18], and deficits of episodic memory are a cognitive hallmark of the disease [19]. Executive dysfunction is evident in the prodromal stage of AD [20–22] and appears predominantly in tasks requiring cognitive flexibility, inhibition, and self-monitoring [23]. There is evidence that the commonly reported impaired ability to perform two tasks simultaneously in AD reflects a specific deficit in dividing attention, rather than the result of a more general processing speed deficit [24]. According to the model of cognitive decline leading to AD presented by Perry and colleagues [25], executive problems appear after memory problems in time, but before typical parietal lobe symptoms (aphasia, visuospatial deficits). When executive dysfunction is present, it has a clear negative influence on ability to manage activities of daily living and may thus add to the risk of conversion from MCI to AD [26]. Predictive accuracy for conversion from MCI to AD for one set of cognitive variables (composed of episodic memory and processing speed measures) has been found to be as high as 0.86 (sensitivity, 0.76; specificity, 0.90) [27]. Gomar et al. [28] found that a test of executive function and assessment of baseline functional capacity predicted conversion from MCI to AD after 2 years better than biomarkers (MR and CSF).

## 3. Executive Nonamnestic MCI

Both aMCI and attention/executive MCI subtypes have been regarded as important predementia subtypes, at risk for AD [29]. While aMCI is usually defined as a prodromal, at-risk condition of AD [30], isolated executive dysfunction can be a prodromal stage for several neurodegenerative diseases. In cases of predementia AD, it is not clear if aMCI and eMCI may be two different categories/subtypes of AD or represent different phases of AD development.

If attention/executive MCI is not a distinct AD subtype, but an earlier stage of AD than aMCI, then aMCI should be expected to have executive/attentional deficits in addition to memory impairment. It has been reported that attention and executive functions may be impaired in the incipient stages of AD and may contribute to the observed memory deficit [31]. It has been argued that even patients defined as “pure” aMCI on screening tests may have executive impairment, when a comprehensive neuropsychological examination of executive cognition is performed [17].

The existence of nonamnestic attention/executive MCI has been recorded in several MCI studies [29, 32–37], where a comprehensive neuropsychological test battery has been utilized for classification purposes. The prevalence of attention/executive MCI will vary widely in different samples based on recruitment criteria and assessment methods but has been reported as from 3 to 15% [38]. In the sample studied by us [37] attention/executive MCI without amnestic deficit constitutes about 30% of the total MCI group, which on the whole is 10–15 years younger than the ADNI study group.

In a study of Johnson and colleagues [35], 31 older adults with pure executive MCI were identified. Of the 12 executive MCI patients who progressed clinically after two years, 2 converted to probable dementia with Lewy bodies, 10 retained the clinical diagnosis of MCI, and none reverted to normal. Patients with single domain executive MCI who progressed quickly over two years had more temporal lobe atrophy on MRI and slightly lower scores for visual memory recall when compared to the stable executive MCI patients, possibly suggesting that converters may be at their later stages of clinical progression. The executive MCI patients who progressed reported fewer dysexecutive symptoms than nonprogressors, while there were no differences in informant-rated dysexecutive symptoms and baseline performance on all four executive tests.

Nine subjects with pure attention/executive MCI were identified in the longitudinal study of Whitwell and colleagues [29]. In this study, almost 70% of MCI patients within an attention/executive subgroup progressed to dementia in the period of four years, suggesting that the group is at high risk of developing dementia. Three patients converted to dementia with Lewy bodies and three patients converted to AD dementia. The prognosis for other patients with isolated attention/executive dysfunction in the study of Whitwell and colleagues is not clear, but they may also convert to other dementias or remain stable over many years.

By using similar criteria for classification of subjects as those used in the study of Whitwell and colleagues, we have identified a bigger group of 23 nonamnestic attention/executive MCI patients [37]. A longitudinal followup will show how many patients will develop AD and other dementias.

## 4. Brain Imaging—MRI Morphometry and Diffusion Tensor Imaging

The attention and executive functions depend on distributed networks [39], encompassing both frontal and parietal associative cortices, as well as subcortical structures and white matter (WM) pathways [40]. The executive functions control and monitor task performance and depend critically on the frontal lobes. Three fronto-subcortical circuits (originating in the prefrontal cortex) have been identified as responsible for executive control functions, that is, the dorsolateral prefrontal cortex (working memory), the lateral orbital cortex (inhibition), and the anterior cingulate cortex (response conflict) [41, 42].

Degeneration of the medial temporoparietal memory network is typical for AD [43]. Findings from functional imaging indicate that during prodromal AD, the brain network involving the dorsolateral prefrontal cortex and the anterior cingulate, is affected [44]. Alterations in these regions have been associated with impairments in executive functions [45]. While aMCI is characterized by medial temporal lobe affection [29], atrophy in the basal forebrain has been found to be characteristic for the MCI groups with isolated attention/executive deficits [29, 33].

Some studies have reported an association between prefrontal cortical changes and attention/executive impairment in MCI [29, 34, 46]. Significant cortical atrophy in frontal regions [47] has been found in predementia AD. It has been argued that prefrontal damage, in combination with cingulate damage, has predictive value for the conversion from MCI to AD [48]. Another recent study indicates that white matter (WM) pathology in AD is distributed in all lobes of the brain but it is most prominent in the frontal WM [49]. In addition to frontal WM changes, MCI patients may have WM changes in both anterior [50] and posterior [51] cingulate regions. The anterior cingulate region is regarded as belonging to a network responsible for executive control function while the posterior cingulate belongs to a memory network [52]. Thus, it has been hypothesized that the caudal portion of the anterior cingulate plays a major role in executive function abilities, primarily through its reciprocal connections with the prefrontal cortex [53, 54].

In our recent study on attention/executive MCI [37], we have demonstrated consistent relationships between neuropsychological function and the microstructural properties of the WM brain pathways measured by diffusion tensor imaging (DTI), as well as cortical-morphometric parameters. Executive impairment in MCI patients with unaffected memory performance has been associated with reduced WM tract integrity (increased radial diffusion (DR) and mean diffusivity (MD)) in frontal and cingulate regions and cortical thinning in caudal middle frontal region. We have found that WM DR/MD increases in frontal, cingulate, and entorhinal regions in patients with attention/executive MCI, but cortical thickness was not different from controls in any of the studied regions [37]. The findings may thus indicate that the relative importance of grey matter versus WM changes may differ at different stages of predementia cognitive impairment [55, 56].

Frontal and temporal WM diffusivity changes have been previously described in aMCI patients [57]. By using DTI to characterize executive networks in MCI, we found WM DR/MD changes in both the anterior and posterior cingulate regions in eMCI suggesting that both regions may contribute to attention/executive impairment in MCI [36]. The cingulate cortex projects into the striatum [42], and both the anterior and posterior cingulate cortices receive mediodorsal thalamic afferents [48], which are part of fronto-subcortical circuits, involved in executive function. Some attention/executive subfunctions correlated significantly with imaging findings in frontal and cingulate regions in the eMCI group, but no significant correlations were found in the controls. In attention/executive MCI, response inhibition was associated with WM DR/MD underlying the superior frontal cortex, and response inhibition/switching was associated with WM DR/MD underlying the superior frontal, rostral middle frontal, lateral/medial orbitofrontal, and retrosplenial cortices. Test scores for attention and divided attention were associated with the cortical thinning of the caudal middle frontal region. The study results thus support the results from previous MCI studies, where associations between prefrontal changes and attention/executive impairment have been reported [29, 34, 46]. In addition, the results confirm that cingulate changes are associated with executive impairment in MCI [48].

In one recent study [34], MCI patients with isolated executive dysfunction had cerebral hypoperfusion in bilateral middle frontal cortex, bilateral posterior cingulated, and the left precuneus relative to controls. Relative to aMCI patients, eMCI patients had hypoperfusion in the left middle frontal cortex, left posterior cingulate, and the left precuneus, supporting the existence of pathophysiologically distinct MCI subgroups.

In the study of Pa and colleagues [33], executive nonamnestic MCI subgroup had significantly less grey matter in the left dorsolateral prefrontal cortex compared with control subjects. The eMCI subgroup had less volume in the caudate nucleus compared with aMCI group, but the differences for prefrontal cortex in eMCI versus aMCI were not significant, which could be due to some reduction of prefrontal cortex volume in aMCI as well. In contrast, the aMCI patients had less volume in the right inferior parietal cortex, typical for AD, than eMCI. These neuroimaging findings also suggest that some of the eMCI patients may represent a distinct subgroup of MCI.

We have found increased entorhinal WM DR and MD in both patients with memory impairment and those with attention/executive dysfunction without objective memory impairment [37, 58], suggesting a common affection of regions known to show changes in early AD. Attention/executive MCI may be an earlier stage of AD than aMCI and the patients with nonamnestic attention/executive impairment may develop memory problems later. It is also possible that patients with attention/executive MCI may progress to non-AD dementias or AD with disproportionate neuropathology in the frontal cortex.

## 5. Nonmemory Findings Associated with Genetic Risk of AD

To study very early development of AD, neurobiological markers of high risk in asymptomatic individuals may be used. Sperling et al. [59] use the term AD-P to denote pathophysiological factors that are significant for the development of clinical AD (AD-C), but each factor in isolation does not cause AD-C. Candidates for markers may be molecular or genetic. Amyloid accumulation may be measured with positron emission tomography (PET) or with cerebrospinal fluid (CSF) analyses, but both are invasive and costly procedures that are not suitable for screening. Genetic risk can be assessed relatively simply, and followup studies of healthy at risk populations are not prohibitively expensive although they have ethical problems.

Apolipoprotein E (APOE) and e4 allele carrier status confer a significant increase in risk of developing AD [60]. Recent studies of relative risk based on large samples [61] argue that the impact of APOE e4 on AD risk is similar to that of major genes in Mendelian diseases and comparable to genetic risk of breast cancer. Amyloid load in cognitively normal persons above age 60 correlates positively with APOE e4 [62]. In MCI patients PIB-positive PET scans are more frequent in APOE e4 carriers [63]. In patients with AD, progression of cerebral amyloid load is associated with e4 gene dose [64]. There is thus evidence that a major genetic risk factor for AD is associated with preclinical accumulation of beta amyloid, the most significant pathophysiological causal factor for developing AD. Greenwood et al. [65] have argued that in view of the complexity of APOE mechanisms affecting cognition, it would be misleading to view all cognitive effects of e4 as evidence of incipient AD, and they argue that in normal aging there is an accumulating effect of inefficient neural repair mechanisms associated with the e4 allele. These changes make the brain more vulnerable to pathological processes, including accumulation of amyloid beta 42 in AD, but do not cause this process to occur in all e4 carriers.

Severe cholinergic changes are found in advanced AD, with loss of cholinergic neurons and receptors [66, 67]. DeKosky et al. [68] found that cholinergic systems are upregulated in MCI individuals. The authors propose that the loss of this apparent compensatory response may mark the conversion of MCI to diagnosable AD. Recent evidence indicates that complex interactions between APOE e4 and cholinergic genes (BuChE) affect the conversion rate of MCI to AD [69] and that the level of beta amyloid accumulation in the brain is related to both APOE and cholinergic activity [70, 71]. Thus we see evidence of a negative interaction between APOE, beta amyloid accumulation, and cholinergic dysfunction.

 There have been numerous studies of cognitive symptoms associated with APOE e4 carrier status in nondemented persons. Wisdom et al. [72] used a meta analysis of more than 2000 participants and found significant positive effect size for memory and global intellectual function. The effect sizes are moderate, and the studies are influenced by choice of methods, especially in nonmemory cognitive domains. Parasuraman and collaborators [73] have taken a cognitive neuroscience approach to study effects of genes involved in risk of AD (APOE) or mechanisms involved in cognitive deficit in AD (cholinergic genes—CHRNA4). They concluded that intact focusing and impaired disengagement of visuospatial attention may be linked to dysfunction in early AD of corticocortical networks linking the posterior parietal and frontal lobes. Greenwood et al. [74] found that healthy middle-aged adults without dementia who carry the APOE e4 allele show deficits in spatial attention and working memory that are qualitatively similar to those seen in clinically diagnosed AD patients. This finding is replicated in an independent sample by Espeseth et al. [75]. The findings support an association between APOE polymorphisms and specific components of visuospatial attention. Later studies have extended the findings to working memory measured by operation span [76]. Greenwood et al. [65] used an experimental paradigm measuring working memory for dot locations in a spatial array and found that accuracy was reduced in healthy e4 homozygotes, of mean age 57–60. Reinvang et al. [77] found that e4 carriers performed worse on letter-number span, another working memory task, and in addition on the Stroop color-word interference task. The groups did not differ in tasks of episodic memory. 

Wishart et al. [78] studied cortical activation pattern in a working memory task and the e4 group showed greater activity during working memory in the medial frontal and parietal regions bilaterally and in the right dorsolateral prefrontal cortex. There were no regions in which the e3 group showed greater activation than the e4 group. By measuring event-related potentials (ERPs) while MCI patients 50–76 years of age worked on an experimental attention task (auditory three-stimulus oddball). Reinvang et al. [77] performed an event related potential (ERP) study with MCI patients and found attenuated N1 and N2 amplitudes in e4 carriers. In a follow up study with only normal controls covering the same age range working on the same auditory oddball task, e4 carriers also had reduced N1 amplitudes. Furthermore, N2 latency was longer for e4 carriers, and this latency predicted memory decline 3.5 years later, suggesting that attention-related functions may presage memory decline in those with elevated risk for AD [80]. The later component P3 has been shown to be associated with APOE in healthy controls. Irimajiri et al. [81] found reduced amplitude among healthy female e4 carriers in auditory task, and Espeseth et al. [82] found e4-related reduction of visual P3a amplitudes. Together, these findings indicate a potential clinical significance of individual differences in the attention-related ERP components N1, N2, and P3. These findings of APOE-related changes in attention are associated with APOE-related differences in brain structure. Espeseth et al. [83] found that healthy e4 carriers had thicker cortices than noncarriers in regions of the brain known to be involved in attentional function. However, an age by APOE interaction showed that this effect was specific for the middle-aged participants. The crosssectional data indicated that there might be an accelerated thinning of the cortex for e4 carriers, suggesting that the thicker cortex among the middle-aged might be associated with a dysfunctional process. Espeseth et al. [82] showed that cortical thickness in regions with significant carrier versus noncarrier diffrerences was negatively correlated with P3a amplitudes, suggesting that the increase in cortical thickness was indeed dysfunctional. Further support for this interpretation was presented by Fortea et al. [84, 85] who showed that while symptomatic PSEN1 mutation carriers had widespread cortical thinning compared to healthy controls, asymptomatic mutation carriers had thicker cortices, suggesting that high risk for AD may be associated with a temporary thickening of the cortex. 

Cognitive functions are sensitive to interaction of APOE with other factors, including interaction with other genes. Espeseth et al. [75] and Reinvang et al. [86] have argued that interaction (epistasis) of APOE and CHRNA4, a nicotinic receptor gene, influences function in the domains of attention and executive function. CHRNA4 has been shown to be related to attentional function in several studies [86–93]. The search for cognitive markers of very early AD, possibly predating amnestic MCI, should therefore take account of the cognitive neuroscience literature on the role of cholinergic systems in attention and executive function. Furthermore, tasks from cognitive neuroscience research that have proven to be related to specific cortical-subcortical activation patterns or neurotransmitter systems may in general be more sensitive to subtle cognitive changes than tests derived from clinical studies of advanced pathology.

## 6. Discussion

Problems of attention and executive function are common in MCI, and in patients with aMCI they are the most important additional symptom domain in multidomain aMCI. It is generally believed that in this group, executive deficit appears after memory impairment in the sequence of cognitive decline leading to full blown dementia.

Executive MCI may occur without memory impairment, and there is evidence that although etiology is heterogenous, a significant proportion of these patients develop AD. How large this group is in MCI samples varies and is dependent on several factors. The strong focus on memory problems as key symptom in early AD, and the wide normal variation in attention and executive function in aging, may indicate a high threshold for these patients to seek medical service. Our own data indicate that in a relatively young MCI population investigated with comprehensive neuropsychological testing, eMCI is a common variant. Attentional and executive dysfunctions may remain undetected even though a thorough neuropsychological examination is performed. Difficulties in dividing attention and manipulating remembered information may be reflected in everyday tasks, such as packing a bag, keeping track of conversations, or walking whilst talking [22]. Patients with executive MCI may show increased behavioral symptoms on questionnaires that specifically measure executive behaviors compared with aMCI and control subjects [33]. Knowing that decreased awareness of cognitive symptoms has been reported in some patients with MCI [94] and executive MCI [95], it may be helpful to ask other informants to rate executive symptoms of the patient.

Current conceptions of early MR changes emphasize hippocampal and cortical atrophy as the significant pathological event, closely linked with emergence of memory impairment [59, 96]. MR analysis yields sensitive measures of a range of pathognomonic events, and recent publications from our group and others indicate that reduced quality of the connectivity of brain networks may compromise cognitive function. This is true, both for the memory network of the brain, including posterior cingulate, and additionally for frontal networks. These networks are involved in both memory and nonmemory functions. Our studies and those of others indicate that in identifying the brain changes underlying eMCI one should emphasize fiber integrity as measures with DTI as well as frontal lobe cortical thinning.

In their report on the status of preclinical markers, AD Sperling and collaborators [59] use the concept of Alzheimer's Disease-pathophysiological process (AD-P) to denote different processes that may contribute to development of clinical Alzheimer's Disease (AD-C). Furthermore they point to studies combining biomarkers (of AD-P) with measures sensitive to very subtle cognitive decline as clearly needed. Large-scale longitudinal studies of biomarker-positive populations raise enormous problems in terms of ethics, costs, and logistics. We suggest that healthy APOE e4 carriers are a realistic and highly relevant study group. Subclinical genetic effects on MR-morphometry [83], DTI [97], and Default Mode [98] have been shown. Experimental cognitive studies have identified specific attention and executive subfunctions as sensitive to APOE allele variation. The paradigms need to be further developed and standardized for use in clinical/epidemiological studies. This development could be modeled on the effort to standardize cognitive neuroscience paradigms for application in schizophrenia research [99]. A great advantage is that they are suited for computerized administration and scoring, so that one may foresee study participants in a longitudinal study logging on to the internet and complete a set of standard tasks at regular intervals.

## References

[1] Albert MS, DeKosky ST, Dickson D (2011). The diagnosis of mild cognitive impairment due to Alzheimer’s disease: recommendations from the National Institute on Aging-Alzheimer’s Association workgroups on diagnostic guidelines for Alzheimer’s disease. *Alzheimer’s and Dementia*.

[2] Winblad B, Palmer K, Kivipelto M, Jelic V, Fratiglioni L (2004). Introduction: mild cognitive impairment: beyond controversies, towards a consensus. *Journal of Internal Medicine*.

[3] Petersen RC, Roberts RO, Knopman DS (2009). Mild cognitive impairment: ten years later. *Archives of Neurology*.

[4] Petersen RC, Aisen PS, Beckett LA (2010). Alzheimer’s Disease Neuroimaging Initiative (ADNI): clinical characterization. *Neurology*.

[5] Petersen RC (1999). Mild cognitive impairment: clinical characterization and outcome. *Archives of Neurology*.

[6] Dubois B (2010). Revising the definition of Alzheimer’s disease: a new lexicon. *The Lancet Neurology*.

[7] Dubois B, Feldman HH, Jacova C (2007). Research criteria for the diagnosis of Alzheimer’s disease: revising the NINCDS-ADRDA criteria. *The Lancet Neurology*.

[8] Rosen HJ, Lengenfelder J, Miller B (2000). Frontotemporal dementia. *Neurologic Clinics*.

[9] Aarsland D, Brønnick K, Fladby T (2011). Mild Cognitive Impairment in Parkinson’s Disease. *Current Neurology and Neuroscience Reports*.

[10] Ferman TJ, Boeve BF, Smith GE (1999). REM sleep behavior disorder and dementia: Cognitive differences when compared with AD. *Neurology*.

[11] Grau-Olivares M, Arboix A (2009). Mild cognitive impairment in stroke patients with ischemic cerebral small-vessel disease: a forerunner of vascular dementia?. *Expert Review of Neurotherapeutics*.

[12] Wilkins CH, Mathews J, Sheline YI (2009). Late life depression with cognitive impairment: evaluation and treatment. *Clinical Interventions in Aging*.

[13] Buckner RL (2004). Memory and executive function in aging and ad: multiple factors that cause decline and reserve factors that compensate. *Neuron*.

[14] Head D, Snyder AZ, Girton LE, Morris JC, Buckner RL (2005). Frontal-hippocampal double dissociation between normal aging and Alzheimer’s disease. *Cerebral Cortex*.

[15] Royall DR, Lauterbach EC, Cummings JL (2002). Executive control function: a review of its promise and challenges for clinical research—a report from the Committee on Research of the American Neuropsychiatric Association. *Journal of Neuropsychiatry and Clinical Neurosciences*.

[16] Miyake A, Friedman NP, Emerson MJ, Witzki AH, Howerter A, Wager TD (2000). The unity and diversity of executive functions and their contributions to complex “frontal lobe” tasks: a latent variable analysis. *Cognitive Psychology*.

[17] Brandt J, Aretouli E, Neijstrom E (2009). Selectivity of executive function deficits in mild cognitive impairment. *Neuropsychology*.

[18] Fratiglioni L, Launer LJ, Andersen K (2000). Incidence of dementia and major subtypes in Europe: a collaborative study of population-based cohorts. *Neurology*.

[19] McKhann GM, Knopman DS, Chertkow H (2011). The diagnosis of dementia due to Alzheimer’s disease: recommendations from the National Institute on Aging-Alzheimer’s Association workgroups on diagnostic guidelines for Alzheimer’s disease. *Alzheimer’s and Dementia*.

[20] Albert M, Moss MB, Blacker D, Tanzi R, McArdle JJ (2007). Longitudinal change in cognitive performance among individuals with mild cognitive impairment. *Neuropsychology*.

[21] Chen P, Ratcliff G, Belle SH, Cauley JA, DeKosky ST, Ganguli M (2000). Cognitive tests that best discriminate between presymptomatic AD and those who remain nondemented. *Neurology*.

[22] Huntley JD, Howard RJ (2010). Working memory in early Alzheimer’s disease: a neuropsychological review. *International Journal of Geriatric Psychiatry*.

[23] Traykov L, Rigaud AS, Cesaro P, Boller F (2007). Neuropsychological impairment in the early Alzheimer’s disease. *Encephale*.

[24] Baddeley AD, Baddeley HA, Bucks RS, Wilcock GK (2001). Attentional control in Alzheimer’s disease. *Brain*.

[25] Perry RJ, Watson P, Hodges JR (2000). The nature and staging of attention dysfunction in early (minimal and mild) Alzheimer’s disease: relationship to episodic and semantic memory impairment. *Neuropsychologia*.

[26] Royall DR, Lauterbach EC, Kaufer D, Malloy P, Coburn KL, Black KJ (2007). The cognitive correlates of functional status: a review from the Committee on Research of the American Neuropsychiatric Association. *Journal of Neuropsychiatry and Clinical Neurosciences*.

[27] Tabert MH, Manly JJ, Liu X (2006). Neuropsychological prediction of conversion to Alzheimer disease in patients with mild cognitive impairment. *Archives of General Psychiatry*.

[28] Gomar JJ (2011). Utility of combinations of biomarkers, cognitive markers, and risk factors to predict conversion from mild cognitive impairment to Alzheimer disease in patients in the Alzheimer’s disease neuroimaging initiative. *Archives of General Psychiatry*.

[29] Whitwell JL, Petersen RC, Negash S (2007). Patterns of atrophy differ among specific subtypes of mild cognitive impairment. *Archives of Neurology*.

[30] Petersen RC (2001). Current concepts in mild cognitive impairment. *Archives of Neurology*.

[31] Storandt M (2008). Cognitive deficits in the early stages of Alzheimer’s disease. *Current Directions in Psychological Science*.

[32] Nordlund A, Rolstad S, Hellström P, Sjögren M, Hansen S, Wallin A (2005). The Goteborg MCI study: mild cognitive impairment is a heterogeneous condition. *Journal of Neurology, Neurosurgery and Psychiatry*.

[33] Pa J, Boxer A, Chao LL (2009). Clinical-neuroimaging characteristics of dysexecutive mild cognitive impairment. *Annals of Neurology*.

[34] Chao LL, Pa J, Duarte A (2009). Patterns of cerebral hypoperfusion in amnestic and dysexecutive MCI. *Alzheimer Disease and Associated Disorders*.

[35] Johnson JK, Pa J, Boxer AL, Kramer JH, Freeman K, Yaffe K (2010). Baseline predictors of clinical progression among patients with dysexecutive mild cognitive impairment. *Dementia and Geriatric Cognitive Disorders*.

[36] Johnson JK, Vogt BA, Kim R, Cotman CW, Head E (2004). Isolated executive impairment and associated frontal neuropathology. *Dementia and Geriatric Cognitive Disorders*.

[37] Grambaite R (2011). Executive dysfunction in mild cognitive impairment is associated with changes in frontal and cingulate white matter tracts. *Journal of Alzheimer's Disease*.

[38] Jak AJ, Bangen KJ, Wierenga CE, Delano-Wood L, Corey-Bloom J, Bondi MW (2009). Contributions of neuropsychology and neuroimaging to understanding clinical subtypes of mild cognitive impairment. *International Review of Neurobiology*.

[39] Reuter-Lorenz PA, Lustig C (2005). Brain aging: reorganizing discoveries about the aging mind. *Current Opinion in Neurobiology*.

[40] Collette F, Hogge M, Salmon E, Van der Linden M (2006). Exploration of the neural substrates of executive functioning by functional neuroimaging. *Neuroscience*.

[41] Cabeza R, Nyberg L (2000). Imaging cognition II: an empirical review of 275 PET and fMRI studies. *Journal of Cognitive Neuroscience*.

[42] Cummings JL (1993). Frontal-subcortical circuits and human behavior. *Archives of Neurology*.

[43] Buckner RL, Wheeler ME (2001). The cognitive neuroscience of remembering. *Nature Reviews Neuroscience*.

[44] Milham MP, Erickson KI, Banich MT (2002). Attentional control in the aging brain: insights from an fMRI study of the stroop task. *Brain and Cognition*.

[45] Bush G, Vogt BA, Holmes J (2002). Dorsal anterior cingulate cortex: a role in reward-based decision making. *Proceedings of the National Academy of Sciences of the United States of America*.

[46] Pa J, Possin KL, Wilson SM (2010). Gray matter correlates of set-shifting among neurodegenerative disease, mild cognitive impairment, and healthy older adults. *Journal of the International Neuropsychological Society*.

[47] Hall AM, Moore RY, Lopez OL, Kuller L, Becker JT (2008). Basal forebrain atrophy is a presymptomatic marker for Alzheimer’s disease. *Alzheimer’s and Dementia*.

[48] Vogt BA (2009). *Cingulate Neurobiology and Disease*.

[49] Sjöbeck M, Elfgren C, Larsson EM (2010). Alzheimer’s disease (AD) and executive dysfunction. A case-control study on the significance of frontal white matter changes detected by diffusion tensor imaging (DTI). *Archives of Gerontology and Geriatrics*.

[50] Mielke MM, Kozauer NA, Chan KCG (2009). Regionally-specific diffusion tensor imaging in mild cognitive impairment and Alzheimer’s disease. *NeuroImage*.

[51] Zhang Y, Schuff N, Jahng GH (2007). Diffusion tensor imaging of cingulum fibers in mild cognitive impairment and Alzheimer disease. *Neurology*.

[52] Buckner RL, Snyder AZ, Shannon BJ (2005). Molecular, structural, and functional characterization of Alzheimer’s disease: evidence for a relationship between default activity, amyloid, and memory. *Journal of Neuroscience*.

[53] Arikuni T, Sako H, Murata A (1994). Ipsilateral connections of the anterior cingulate cortex with the frontal and medial temporal cortices in the macaque monkey. *Neuroscience Research*.

[54] Vogt BA, Crino PB, Volicer L (1991). Laminar alterations in *γ*-aminobutyric acid(A), muscarinic, and *β* adrenoceptors and neuron degeneration in cingulate cortex in Alzheimer’s disease. *Journal of Neurochemistry*.

[55] Jack CR, Lowe VJ, Weigand SD (2009). Serial PIB and MRI in normal, mild cognitive impairment and Alzheimers disease: implications for sequence of pathological events in Alzheimers disease. *Brain*.

[56] De la Monte SM (1989). Quantitation of cerebral atrophy in preclinical and end-stage Alzheimer’s disease. *Annals of Neurology*.

[57] Wang L, Goldstein FC, Veledar E (2009). Alterations in cortical thickness and white matter integrity in mild cognitive impairment measured by whole-brain cortical thickness mapping and diffusion tensor imaging. *American Journal of Neuroradiology*.

[58] Grambaite R, Reinvang I, Selnes P (2011). Pre-dementia memory impairment is associated with white matter tract affection. *Journal of the International Neuropsychological Society*.

[59] Sperling RA, Aisen PS, Beckett LA (2011). Toward defining the preclinical stages of Alzheimer’s disease: recommendations from the National Institute on Aging-Alzheimer’s Association workgroups on diagnostic guidelines for Alzheimer’s disease. *Alzheimer’s and Dementia*.

[60] Corder EH, Saunders AM, Strittmatter WJ (1993). Gene dose of apolipoprotein E type 4 allele and the risk of Alzheimer’s disease in late onset families. *Science*.

[61] Genin E (2011). APOE and Alzheimer disease: a major gene with semi-dominant inheritance. *Molecular Psychiatry*.

[62] Caselli RJ, Walker D, Sue L, Sabbagh M, Beach T (2010). Amyloid load in nondemented brains correlates with APOE e4. *Neuroscience Letters*.

[63] Pike KE, Savage G, Villemagne VL (2007). *β*-amyloid imaging and memory in non-demented individuals: evidence for preclinical Alzheimer’s disease. *Brain*.

[64] Grimmer T, Tholen S, Yousefi BH (2010). Progression of cerebral amyloid load is associated with the apolipoprotein e *ε*4 genotype in Alzheimer’s disease. *Biological Psychiatry*.

[65] Greenwood PM, Sunderland T, Lambert C, Parasuraman R (2005). Effects of apolipoprotein E genotype on spatial attention, working memory, and their interaction in healthy, middle-aged adults: results from the National Institute of Mental Health’s BIOCARD study. *Neuropsychology*.

[66] Davies P, Maloney AJF (1976). Selective loss of central cholinergic neurons in Alzheimer’s disease. *The Lancet*.

[67] Whitehouse PJ, Price DL, Struble RG (1982). Alzheimer’s disease and senile dementia: loss of neurons in the basal forebrain. *Science*.

[68] Dekosky ST, Ikonomovic MD, Styren SD (2002). Upregulation of choline acetyltransferase activity in hippocampus and frontal cortex of elderly subjects with mild cognitive impairment. *Annals of Neurology*.

[69] Lane R, Feldman HH, Meyer J (2008). Synergistic effect of apolipoprotein E *ε*4 and butyrylcholinesterase K-variant on progression from mild cognitive impairment to Alzheimer’s disease. *Pharmacogenetics and Genomics*.

[70] Darreh-Shori T, Forsberg A, Modiri N (2011). Differential levels of apolipoprotein E and butyrylcholinesterase show strong association with pathological signs of Alzheimer’s disease in the brain in vivo. *Neurobiology of Aging*.

[71] Bao F, Wicklund L, Lacor PN, Klein WL, Nordberg A, Marutle A (2012). Different *β*-amyloid oligomer assemblies in Alzheimer brains correlate with age of disease onset and impaired cholinergic activity. *Neurobiology of Aging*.

[72] Wisdom NM, Callahan JL, Hawkins KA (2011). The effects of apolipoprotein E on non-impaired cognitive functioning: a meta-analysis. *Neurobiology of Aging*.

[73] Parasuraman R, Greenwood PM, Haxby JV, Grady CL (1992). Visuospatial attention in dementia of the Alzheimer type. *Brain*.

[74] Greenwood PM, Sunderland T, Friz JL, Parasuraman R (2000). Genetics and visual attention: selective deficits in healthy adult carriers of the *ε*4 allele of the apolipoprotein E gene. *Proceedings of the National Academy of Sciences of the United States of America*.

[75] Espeseth T, Greenwood PM, Reinvang I (2006). Interactive effects of APOE and CHRNA4 on attention and white matter volume in healthy middle-aged and older adults. *Cognitive, Affective and Behavioral Neuroscience*.

[76] Rosen VM, Bergeson JL, Putnam K, Harwell A, Sunderland T (2002). Working memory and apolipoprotein E: what’s the connection?. *Neuropsychologia*.

[77] Reinvang I, Winjevoll IL, Rootwelt H, Espeseth T (2010). Working memory deficits in healthy APOE epsilon 4 carriers. *Neuropsychologia*.

[78] Wishart HA, Saykin AJ, Rabin LA (2006). Increased brain activation during working memory in cognitively intact adults with the APOE *ε*4 allele. *American Journal of Psychiatry*.

[79] Reinvang I, Espeseth T, Gjerstad L (2005). Cognitive ERPs are related to ApoE allelic variation in mildly cognitively impaired patients. *Neuroscience Letters*.

[80] Espeseth T, Rootwelt H, Reinvang I (2009). Apolipoprotein E modulates auditory event-related potentials in healthy aging. *Neuroscience Letters*.

[81] Irimajiri R, Golob EJ, Starr A (2010). ApoE genotype and abnormal auditory cortical potentials in healthy older females. *Neurobiology of Aging*.

[82] Espeseth T, Westlye LT, Walhovd KB (2012). Apolipoprotein E *ε*4-related thickening of the cerebral cortex modulates selective attention. *Neurobiology of Aging*.

[83] Espeseth T, Westlye LT, Fjell AM, Walhovd KB, Rootwelt H, Reinvang I (2008). Accelerated age-related cortical thinning in healthy carriers of apolipoprotein E *ε*4. *Neurobiology of Aging*.

[84] Fortea J, Sala-Llonch R, Bartrés-Faz D (2010). Increased cortical thickness and caudate volume precede atrophy in psen1 mutation carriers. *Journal of Alzheimer’s Disease*.

[85] Fortea J, Sala-Llonch R, Bartrés-Faz D (2011). Cognitively preserved subjects with transitional cerebrospinal fluid ß-amyloid 1-42 values have thicker cortex in Alzheimer’s disease vulnerable areas. *Biological Psychiatry*.

[86] Reinvang I, Lundervold AJ, Rootwelt H, Wehling E, Espeseth T (2009). Individual variation in a cholinergic receptor gene modulates attention. *Neuroscience Letters*.

[87] Espeseth T, Endestad T, Rootwelt H, Reinvang I (2007). Nicotine receptor gene CHRNA4 modulates early event-related potentials in auditory and visual oddball target detection tasks. *Neuroscience*.

[88] Espeseth T, Sneve MH, Rootwelt H, Laeng B (2010). Nicotinic receptor gene chrna4 interacts with processing load in attention. *PLoS ONE*.

[89] Greenwood PM, Fossella JA, Parasuraman R (2005). Specificity of the effect of a nicotinic receptor polymorphism on individual differences in visuospatial attention. *Journal of Cognitive Neuroscience*.

[90] Greenwood PM, Lin MK, Sundararajan R, Fryxell KJ, Parasuraman R (2009). Synergistic effects of genetic variation in nicotinic and muscarinic receptors on visual attention but not working memory. *Proceedings of the National Academy of Sciences of the United States of America*.

[91] Greenwood PM, Sundararajan R, Lin MK, Kumar R, Fryxell KJ, Parasuraman R (2009). Both a nicotinic single nucleotide polymorphism (SNP) and a noradrenergic SNP modulate working memory performance when attention is manipulated. *Journal of Cognitive Neuroscience*.

[92] Winterer G, Musso F, Konrad A (2007). Association of attentional network function with exon 5 variations of the CHRNA4 gene. *Human Molecular Genetics*.

[93] Parasuraman R, Greenwood PM, Kumar R, Fossella J (2005). Beyond heritability: neurotransmitter genes differentially modulate visuospatial attention and working memory. *Psychological Science*.

[94] Vogel A, Hasselbalch SG, Gade A, Ziebell M, Waldemar G (2005). Cognitive and functional neuroimaging correlates for anosognosia in mild cognitive impairment and Alzheimer’s disease. *International Journal of Geriatric Psychiatry*.

[95] Johnson JK, Pa J, Boxer AL, Kramer JH, Freeman K, Yaffe K (2010). Baseline predictors of clinical progression among patients with dysexecutive mild cognitive impairment. *Dementia and Geriatric Cognitive Disorders*.

[96] Jack CR, Knopman DS, Jagust WJ (2010). Hypothetical model of dynamic biomarkers of the Alzheimer’s pathological cascade. *The Lancet Neurology*.

[97] Heise V, Filippini N, Ebmeier KP, Mackay CE (2010). The APOE epsilon 4 allele modulates brain white matter integrity in healthy adults. *Molecular Psychiatry*.

[98] Filippini N (2009). Distinct patterns of brain activity in young carriers of the APOE-epsilon4 allele. *Proceedings of the National Academy of Sciences of the United States of America*.

[99] Carter CS, Barch DM (2007). Cognitive neuroscience-based approaches to measuring and improving treatment effects on cognition in schizophrenia: the CNTRICS initiative. *Schizophrenia Bulletin*.

